# After the Liverpool Care Pathway—development of heuristics to guide end of life care for people with dementia: protocol of the ALCP study

**DOI:** 10.1136/bmjopen-2015-008832

**Published:** 2015-09-02

**Authors:** N Davies, J Manthorpe, E L Sampson, S Iliffe

**Affiliations:** 1 Research Department of Primary Care & Population Health, UCL, London, UK; 2 Social Care Workforce Research Unit, Kings College London, London, UK; 3 Marie Curie Palliative Care Research Department, Division of Psychiatry, University College London, London, UK; 4 Barnet Enfield and Haringey Mental Health Trust Liaison Team, North Middlesex University Hospital, London, UK

**Keywords:** PALLIATIVE CARE, end of life care, decision making, heuristic

## Abstract

**Introduction:**

End of life care guidance for people with dementia is lacking and this has been made more problematic in England with the removal of one of the main end of life care guidelines which offered some structure, the Liverpool Care Pathway. This guidance gap may be eased with the development of heuristics (rules of thumb) which offer a fast and frugal form of decision-making.

**Objective:**

To develop a toolkit of heuristics (rules of thumb) for practitioners to use when caring for people with dementia at the end of life.

**Method and analysis:**

A mixed-method study using a co-design approach to develop heuristics in three phases. In phase 1, we will conduct at least six focus groups with family carers, health and social care practitioners from both hospital and community care services, using the ‘think-aloud’ method to understand decision-making processes and to develop a set of heuristics. The focus group topic guide will be developed from the findings of a previous study of 46 interviews of family carers about quality end-of-life care for people with dementia and a review of the literature. A multidisciplinary development team of health and social care practitioners will synthesise the findings from the focus groups to devise and refine a toolkit of heuristics. Phase 2 will test the use of heuristics in practice in five sites: one general practice, one community nursing team, one hospital ward and two palliative care teams working in the community. Phase 3 will evaluate and further refine the toolkit of heuristics through group interviews, online questionnaires and semistructured interviews.

**Ethics and dissemination:**

This study has received ethical approval from a local NHS research ethics committee (Rec ref: 15/LO/0156). The findings of this study will be presented in peer-reviewed publications and national and international conferences.

**Strengths and limitations of this study**

- This study places a high emphasis on family carers and will utilise their experience and knowledge to develop heuristics through a process of co-design.
- Heuristics offer a novel approach to decision-making at the end of life.
- The heuristics developed in this study will be tested in a range of settings.

## Introduction

At present, no treatment can alter the course of any form of dementia. Estimated average survival from when the person with dementia first notices symptoms is between 4 and 5 years^1^ and from receiving a diagnosis is 3.5 years.^2^ End-of-life care is therefore rapidly becoming one of the major priorities for dementia care. There are currently more than 670 000 family members and friends caring for people with dementia in the UK.^3^ These carers often provide the majority of health and social care, especially earlier in the course of dementia, and without them the professional health and care systems would be likely to collapse.^4^

In England, the government's End of Life Care Strategy defines end-of-life care as the last 12 months of life,^5^ but this can be a problem when supporting people with dementia and those caring for them because it is often not possible to know how their dementia will progress and how other illness may affect the dementia.^6^ For this study, the researchers take the view that end-of-life care is not a period of time limited to the final days, hours or weeks of life, but more a period when the person, their family or practitioners recognise that they are dying^7^ and this will vary for individuals.

Guidance for practitioners on end-of-life care in England is currently specific guidance only for end-of-life care for people with cancer and not for other conditions, such as dementia.^8^ One of the main documents referred to by practitioners at the end of life was the Liverpool Care Pathway which offered a way for practitioners to plan care for someone who was at the very end of their life, often the final 48 h. The Liverpool Care Pathway involved the withdrawal of unnecessary medication and interventions, and emphasised attention to the personal needs of the dying patient.

Growing media attention highlighted concern about the ways in which end-of-life care was being delivered within England. This culminated in an independent review of the Liverpool Care Pathway.^9^ As a consequence, the government announced that it would gradually phase out the Liverpool Care Pathway. Many of the media reports were from family members who felt their older relatives were abandoned or treated differently because of their age. Practitioners’ criticisms of the failings of the Liverpool Care Pathway included its overemphasis on ‘paper work’ which led to a lack of attention to care. Many considered that these problems were exacerbated by a misinterpretation of many of the key features of the Liverpool Care Pathway, including nutrition, and hydration, together with a lack of training about its implementation.^9–11^

Some practitioners argue that quality care for people with dementia at the end of life is inhibited by its lack of clear structure.^12^ The Liverpool Care Pathway provided some support and a structure to guide care practitioners. Its withdrawal following the public criticisms has resulted in a potential ‘guidance gap’ as well as a potential decline in confidence among practitioners.^13^ ^14^ The removal of the Liverpool Care Pathway coincided with the publication of the European Association for Palliative Care's white paper which defined optimal palliative care for people with dementia and their families.^15^ This potentially provides the first set of guidance specific for dementia end-of-life care for practitioners but lacks policy endorsement and the controversy over the Liverpool Care Pathway may affect its reception.

Robust scientific conclusions derived from randomised controlled trials or epidemiological studies are too scarce to inform practitioners’ decision-making in many areas of practice and many guidelines are not sufficiently based on evidence and are of low quality.^16^ Instead, heuristics (‘rules of thumb’ or ‘mental short-cuts’) are widely employed to address everyday problems.^17^ Typically, heuristics are used in situations of uncertainty, may rely on first impressions, and can occur effortlessly as a form of fast and frugal decision-making that frequently gets the right answer.^18^ However, they are also prone to multiple biases and can easily provide the wrong answer; they are assumed by many in healthcare to give second-best outcomes.^19^ Nevertheless, heuristics may well be the only solution to managing poorly defined problems where no robust evidence exists and speedy decisions are needed. When uncertainty is high, decision-makers need to use the minimal amount of relevant information—in these circumstances, less is more.^20^ Fast and frugal heuristics have also been shown to be more accurate than more complex and sophisticated prediction tools.^21^ Heuristics are important to practise, but to reduce errors and avoid biases they should be discussed, criticised, refined and taught.^17^

‘FAST’ is an example of a well-known heuristic designed to guide responses to stroke symptoms (standing for Facial drooping, Arm weakness, Speech difficulties, Time to call emergency services). FAST has demonstrated increased accuracy of the identification of patients with stroke.^22^ Similarly, PAID may be useful for practitioners trying to understand the causes of challenging behaviour in people with dementia (standing for Pain, Aggravation, Intrinsic to dementia (eg, wandering), Depression/Delusions). The heuristics that general practitioners use in making clinical decisions appear to shape performance more powerfully than any form of formal training.^23^

End-of-life care for dementia can be very difficult for many reasons, not least because of the difficulty in communicating verbally which many people with dementia have towards the end of life. Many practitioners, both those from palliative care backgrounds and those with experience in dementia care, lack the confidence and skills to provide end-of-life care for someone with dementia.^24^ This includes practitioners working in care homes where resources, staffing levels and regulatory requirements are limited, and support from the wider healthcare system is variable. End-of-life care for people with dementia is for these reasons often poor, with improvement needed in many areas.^25^ There is still limited access to end of life care services for people with dementia,^5^ ^26^ ^27^ with a lack of the recognition of pain often highlighted,^28^ ^29^ some even believing that people with dementia do not experience pain.^30^

The challenge remains how best to improve end-of-life care in the light of the recent Liverpool Care Pathway review, family and practitioner anxieties and media controversy.^9^ One critically important resource is those people close to the person with dementia, often family members. However, rarely have the views and experiences of family carers in their own right been elicited^31^ and little is known about the experiences of carers about end-of-life care.^26^ ^31^ ^32^ The recent descriptions of poor end of life care surrounding the Liverpool Care Pathway have created an urgent need for health and social care practitioners to make more use of the experiences of families, some of whom experience the dilemmas of care on a daily basis.

The removal of the Liverpool Care Pathway has left a gap in the guidance for practitioners which may need to be filled, as suggested by claims that some organisations are finding it hard to adapt to the Pathway's removal and suspicions that some are simply using it under a different name.^33^ We have proposed that this gap could be filled with the assistance of the families of people with dementia, some of whose experiences were similar to those that brought about the demise of the Liverpool Care Pathway. There remains little practice related training in end-of-life care for people with dementia, with dementia apparently still often not being accepted as an illness which will lead to death, sometimes requiring specialist end of life care input.^34^

Starting with data from 46 interviews with family carers about end-of-life care for people with dementia as a foundation^35^(collected and funded as part of the IMPACT study^36^), our three-phase study described in this paper aims to:

1. Conduct focus groups with family carers both current and former, as well as health and social care practitioners to understand decision-making at the end of life. Data collected from the focus groups, literature and the previous interviews will be synthesised to produce heuristics. These novel heuristics will be discussed, criticised and refined in an iterative process involving experts by training and experts by experience, as recommended by McDonald.^17^
2. Test the use of heuristics with practitioners in five real settings including: one general practice, one community nursing team, one care of the elderly hospital ward and two community palliative care teams.
3. Evaluate the use content, and further refine the toolkit of heuristics through individual interviews, group interviews and online questionnaires.

## Methods and analysis

### Design

**Figure 1:**
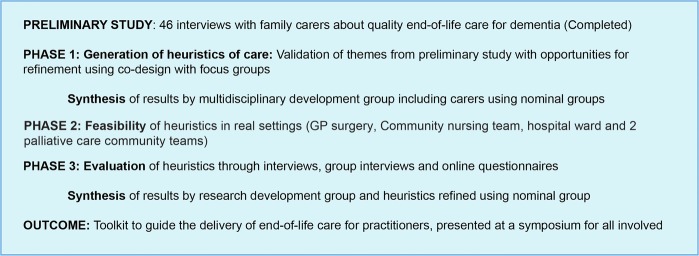
Overview of the project.

This study will use mixed methods and comprise three phases: phase 1 will use input from interviews with families plus findings from focus groups with families and practitioners, and a rapid appraisal literature review to develop a collection of heuristics; phase 2 will test the feasibility of the developed heuristics in real settings and phase 3 will evaluate the heuristics using semistructured interviews and group interviews, finishing by synthesising the learning from all sites to create the final heuristics. Figure 1 shows the project's flow path.

We will develop the heuristics using a standard, well-established developmental approach for creating decision support guides.^37^ We have successfully used this method to develop a decision support system for dementia diagnosis and management,^38^ ^39^ which was incorporated into the electronic medical records system EMIS (Egton medical Informatics System) after being shown to improve practice in a randomised controlled trial.^40^ A co-design approach^41^ will be used to engage carers and practitioners in the identification of important aspects of end-of-life care where heuristics might usefully be applied and the subsequent operationalisation of the heuristics.

Focus groups will be organised for carers and practitioners from different disciplines and the interaction within groups will be facilitated to promote professional creativity and debate around the usefulness and effectiveness of individual heuristics.^42^

The findings from the focus groups and literature reviews will be synthesised, developed and refined into heuristics using a modified nominal group technique with a development group. Nominal groups are potentially powerful learning and development tools.^43^ A nominal group process is a structured meeting which seeks to facilitate group or team decision-making about a given problem (generation of heuristics) from a group who are experts in the given field. The process involves an introduction from the facilitator, silent generation of ideas by individuals, group discussion of generated ideas and ranking of ideas to the problem being discussed (in this case, heuristics).^44^ They have a particularly useful role in analysing healthcare problems,^44^ and can help bridge the gap between researchers and practitioners.^45^ A nominal group approach designed for ill-structured problems will be used, to allow for disagreements over problem definition, and to produce potential solutions that overlap or vary widely in specificity. This will require the synthesis process to generate ideas, confirm that it is addressing the same problem, analyse the content of the heuristics and categorise and clarify them.^46^

### Participants and settings

The focus of the study is on end-of-life care for people with dementia in their own homes, acknowledging that some may also classify a care home as their home (community), and on end-of-life care in general hospitals.

*Phase 1 will aim to recruit:*

1. Former family carers and family carers who are currently caring for someone with dementia;
2. Practitioners working with people with dementia at the end of life, including admiral nurses (specialist dementia nurses), general practitioners, community nurses, hospital nurses, healthcare assistants, palliative care teams, social workers, psychiatrists, psychologists and geriatricians.

*Phases 2 and 3 will take place in:*

1. One care of the elderly hospital ward;
2. One general practice;
3. One community nursing team;
4. Two palliative care community teams.

### Recruitment

Family carers for the first phase will be recruited through the Alzheimer's Society and other carers’ organisations, such as a local Carers Service and the Carers Trust. We will also utilise the Patient and Public Involvement Forum and the clinical studies groups of the Dementias and Neurodegenerative Diseases Research Network (DeNDRoN) and the North Thames Dementia Registry. We will seek help from the DeNDRoN co-ordinating centre, the Comprehensive Local Research Network (CLRN), the education sector and the Central North West London NHS Foundation Trust for recruitment of practitioners and social care employers in the same localities. For the second phase of the study, we will make use of the above networks to recruit a community nursing team, a general practice which has care home responsibilities and a hospital ward. We will seek advice from Marie Curie and members of the research team who have experience of successfully recruiting palliative care teams. All participants will receive a verbal explanation of the study as well as a written copy of the information sheet, and will be given the opportunity to ask any questions. An experienced researcher (ND) will collect informed consent from all participants and the lead site manager/director prior to study participation.

### Procedure

#### Phase 1: development and generation of heuristics using focus groups based on data from 46 interviews

At least six focus groups will be conducted with carers, practitioners and other experts. Family carers will be offered individual interviews if preferred. The focus groups will last between 1 and 1.5 h and will be facilitated by a researcher with experience of group facilitation to include:

- Group 1: Up to five bereaved family carers discussing heuristics for hospital care.
- Group 2: Up to five carers currently caring for someone with dementia discussing heuristics for hospital care.
- Group 3: Five–eight practitioners involved in end-of-life care for people with dementia in hospital discussing heuristics for hospital care.
- Group 4: Up to five bereaved family carers discussing heuristics for home care.
- Group 5: Up to five carers currently caring for someone with dementia discussing heuristics for home care.
- Group 6: Five–eight practitioners involved in end-of-life care for people with dementia at home discussing heuristics for home care.

The focus groups will use the ‘think aloud’ method^47^ which encourages people to vocalise their thought process when performing tasks or solving problems. Many have argued that attention is needed for verbalisation of thought processes as this highlights an individual's cognitive–behaviour and information stored in working memory.^47–49^

Each group will be invited to follow the same four stage procedure to discuss and devise heuristics:

- Stage 1: Introduction (5 min)—an introduction from the facilitator, explaining the purpose of the study and the focus groups.
- Stage 2: Opening the topic (5 min)—the facilitator will introduce quality of care ideas developed from the results of 46 in depth interviews (already conducted) with family carers and a review of the literature.
- Stage 3: Discussion (think-aloud) (up to 70 min)—six topics of quality end of care and/or possible topics of heuristics will be displayed on a screen individually for up to 10 min each and participants will be asked to discuss, think about their experience with this topic, what decisions need to be made, and finally what are the right decisions, while verbalising their thought processes. The facilitator will record key ideas on a flip chart.
- Stage 4: Summary and close (10 min)—the facilitator will round up the group discussion with a summary of the key topics and thoughts from the group.

A separate development group consisting of ten health and social care practitioners, as well as family carer representatives, will be formed and begin to meet regularly after the first focus group. They will assist with the synthesis of the results and construction of a set of heuristics, acting as a think tank and providing a validation process using a nominal group process as described above.

#### Phase 2: feasibility of heuristics

Practitioners will be asked to use the heuristics as a framework and basis when providing end-of-life care for up to 10 people with dementia for a period of 6 months in each setting.

#### Phase 3: evaluation of heuristics

##### Three-month questionnaire and group interviews

Up to five practitioners from each site will be asked to complete an online questionnaire about their use of the heuristics. This will act as an early indication of the use of heuristics and will enable the research team to identify and address any major concerns, and reinforce the use of heuristics to guide care. We will also undertake group interviews at each site consisting of 5–8 participants, with practitioners to gain a better understanding of whether the heuristics are working and, if not, why. Group interviews keep staff time to a minimum and collect a variety of ideas while allowing for interaction and discussion of these ideas. They will be conducted using nominal group methods (see above).

##### Six-month interviews

Following phase 2, semistructured interviews will be conducted with the use of a topic guide on a one-to-one basis with practitioners (5–8 per site) who have applied the heuristics in practice. This will inform the final iterations of the heuristics with the research development team.

### Analysis

Interviews will be transcribed verbatim and thematic analysis methods will be used to analyse the data. Coding will be led by one researcher and checked by two further researchers who will meet regularly to discuss emerging themes to enhance reliability and rigour.^50–52^ The development group will be convened and invited to discuss the evaluation and discuss the final set of heuristics using nominal group procedures.

## Ethics and dissemination

The output of this study will be a series of heuristics (rules of thumb) developed using carers’ experiences and views, as well as practitioner experiences and opinions, which have been tested with practitioners caring for people with dementia at end of life in various settings.

The findings from this study will be presented in peer-reviewed journals both within palliative care and dementia care journals to target a wide audience which this study will be relevant for. Findings will be presented at national and international conferences, and professional press such as *Journal of Dementia Care* will be utilised to increase the spread of knowledge generated. Finally, a study website will be developed and social media such as twitter and blogs will be used to disseminate findings.

## Contribution to knowledge and practice

The diversity of end of life care provision has prompted a search for a common language to describe it,^7^ while there is greater acknowledgement of the importance of capturing the complexities of provision.^53^ End-of-life care for people with cancer is relatively well developed, in terms of its conceptual framework and evidence base.^54^ The evidence base to guide practice in end-of-life care for people with dementia is less well developed, although it is now evolving.^55^ This study will contribute to the common language, and to the development of practice. The heuristics it develops and tests may help fill the gap left by the departure of the Liverpool Care Pathway.

## References

[1] Xie J, Brayne C, Matthews FE Survival times in people with dementia: analysis from population based cohort study with 14 year follow-up. Br Med J 2008;336:258–62. 10.1136/bmj.39433.616678.25 18187696 PMC2223023

[2] Rait G, Walters K, Bottomley C Survival of people with clinical diagnosis of dementia in primary care: cohort study. Br Med J 2010;341:c3584 10.1136/bmj.c3584 20688840 PMC2917003

[3] Alzheimer's Society. *Dementia 2012: A national challenge*. London: Alzheimer's Society, 2012. http://www.alzheimers.org.uk/site/scripts/download_info.php?fileID=1389 (accessed May 2015)

[4] Alzheimer's Society. Carer Support. Secondary Carer Support 2013. http://www.alzheimers.org.uk/site/scripts/documents_info.php?documentID=546

[5] Department of Health. End of life care strategy: promoting high quality care for all adults at the end of life. London: Department of Health, 2008.

[6] Sachs GA, Shega JW, Cox-Hayley D Barriers to excellent end-of-life care for patients with dementia. J Gen Intern Med 2004;19:1057–63. 10.1111/j.1525-1497.2004.30329.x PMC1492583 15482560

[7] Radbruch L, Payne S White paper on standards and norms for hospice and palliative care in Europe: part 1. European Journal of Palliative Care 2009;16:278–89.

[8] National Institute for Health and Care Excellence. Improving supportive and palliative care for adults with cancer. National Institute for Health and Care Excellence (NICE), London, 2004.

[9] Neuberger J More care, less pathway: a review of the liverpool care pathway. Department of Health, London, 2013.

[10] Chinthapalli K The Liverpool care pathway: what do specialists think? BMJ 2013;346:f1184 10.1136/bmj.f1184 23449644

[11] Chinthapalli K The birth and death of the Liverpool care pathway. Br Med J 2013;347:f4669 10.1136/bmj.f4669 23884973

[12] Harrison-Dening K, Greenish W, Jones L Barriers to providing end-of-life care for people with dementia: a whole-system qualitative study. BMJ Support Palliat Care 2012;2:103–7. 10.1136/bmjspcare-2011-000178 24654049

[13] McCartney M The assault on the Liverpool care pathway. BMJ 2012;345:e7316 10.1136/bmj.e7316 23112122

[14] Torjesen I Bad press over Liverpool care pathway has scared patients and doctors, say experts. Br Med J 2013;346:f175 10.1136/bmj.f175 23305844

[15] van der Steen JT, Radbruch L, Hertogh CM White paper defining optimal palliative care in older people with dementia: a Delphi study and recommendations from the European Association for Palliative Care. Palliat Med 2014;28:197–209. 10.1177/0269216313493685 23828874

[16] Grol R Improving the quality of medical care: building bridges among professional pride, payer profit, and patient satisfaction. JAMA 2001;286:2578–85. 10.1001/jama.286.20.2578 11722272

[17] McDonald CJ Medical heuristics: the silent adjudicators of clinical practice. Ann Intern Med 1996;124:56–62. 10.7326/0003-4819-124-1_Part_1-199601010-00009 7503478

[18] Croskerry P A universal model of diagnostic reasoning. Acad Med 2009;84:1022–8. 10.1097/ACM.0b013e3181ace703 19638766

[19] Elstein AS Heuristics and biases: selected errors in clinical reasoning. Acad Med 1999;74:791–4. 10.1097/00001888-199907000-00012 10429587

[20] Wegwarth O, Gaissmaier W, Gigerenzer G Smart strategies for doctors and doctors-in-training. Med Educ 2009;43:721–8. 10.1111/j.1365-2923.2009.03359.x 19573016

[21] Marewski JN, Gigerenzer G Heuristic decision making in medicine. Dialogues Clin Neurosci 2012;14:77–89. 22577307 10.31887/DCNS.2012.14.1/jmarewski PMC3341653

[22] Harbison J, Hossain O, Jenkinson D Diagnostic accuracy of stroke referrals from primary care, emergency room physicians, and ambulance staff using the face arm speech test. Stroke 2003;34:71–6. 10.1161/01.STR.0000044170.46643.5E 12511753

[23] Andersson SJ, Lindberg G, Troein M What shapes GPs’ work with depressed patients? A qualitative interview study. Fam Pract 2002;19:623–31. 10.1093/fampra/19.6.623 12429665

[24] Davies N, Maio L, Vedavanam K Barriers to the provision of high quality palliative care for people with dementia in England: a qualitative study of professionals’ experiences. Health Soc Care Community 2014;22:386–94. 10.1111/hsc.12094 24372976 PMC4265301

[25] Dixon J, King D, Matosevic T Equity in the provision of palliative care in the UK: review of evidence. London: Marie Curie, 2015. https://www.mariecurie.org.uk/globalassets/media/documents/policy/campaigns/equity-palliative-care-uk-report-full-lse.pdf (accessed May 2015)

[26] Sampson EL Palliative care for people with dementia. Br Med Bull 2010;96:159–74. 10.1093/bmb/ldq024 20675657

[27] Banerjee S, Owen J Living well with dementia: a national dementia strategy. London: Department of Health, 2009.

[28] Scherder E, Oosterman J, Swaab D Recent developments in pain in dementia. BMJ 2005;330:461–4. 10.1136/bmj.330.7489.461 15731144 PMC549660

[29] Sampson EL, Gould V, Lee D Differences in care received by patients with and without dementia who died during acute hospital admission: a retrospective case note study. Age Ageing 2006;35:187–9. 10.1093/ageing/afj025 16407434

[30] Davies N, Maio L, Van Riet Paap J Quality palliative care for cancer and dementia in five European countries: some common challenges. Aging Ment Health 2013;18:400–10. 10.1080/13607863.2013.843157 24131061 PMC3979441

[31] Davies N, Maio L, Rait G Quality end-of-life care for dementia: What have family carers told us so far? A narrative synthesis. Palliat Med 2014;28:919–30. 10.1177/0269216314526766 24625567 PMC4232347

[32] Raymond M, Warner A, Davies N Palliative care services for people with dementia: a synthesis of the literature reporting the views and experiences of professionals and family carers. Dementia 2014;13:96–110. 10.1177/1471301212450538 24381041

[33] O'Dowd A Banned end of life pathway is still in use under another name, MPs hear. *BMJ* 2015;350:h555. 10.1136/bmj.h555 25633428

[34] Burns A, Wee B The challenges of end-of-life care in people wiht dementia. Eur J Pallitative Care 2015;22:57.

[35] Davies N Talking with family carers about end-of-life care for people with dementia. Eur J Palliative Care 2015;22:6–8.

[36] Iliffe S, Davies N, Vernooij-Dassen M Modelling the landscape of palliative care for people with dementia: a European mixed methods study. BMC Palliat Care 2013;12:30(30). 10.1186/1472-684X-12-30 PMC3751306 23937891

[37] Wyatt J, Spiegelhalter D Evaluating medical expert systems: what to test and how? Med Inform (Lond) 1990;15:205–17. 10.3109/14639239009025268 2232956

[38] Iliffe S, Austin T, Wilcock J Design and implementation of a computer decision support system for the diagnosis and management of dementia syndromes in primary care. Methods Inf Med 2002;41:98–104. 12061130

[39] Turner S, Iliffe S, Downs M Decision support software for dementia diagnosis and management in primary care: relevance and potential. Aging Ment Health 2003;7:28–33. 10.1080/1360789021000058148 12554312

[40] Downs M, Turner S, Bryans M Effectiveness of educational interventions in improving detection and management of dementia in primary care: cluster randomized controlled study. BMJ 2006;332:692–6. 10.1136/bmj.332.7543.692 16565124 PMC1410839

[41] Kaulio MA Customer, consumer and user involvement in product development: a framework and a review of selected methods. Total Qual Manage 1998;9:141–9. 10.1080/0954412989333

[42] Barbour R Introducing qualitative research: a students guide. London: Sage, 2014.

[43] Dockery G Rhetoric or reality? Participatory research in the National Health service, UK. In: De Koning K, Martin M, eds. Participatory research in health: issues and experiences. London: Zed books, 1996:164–76.

[44] Van de Ven AH, Delbecq AL The nominal group as a research instrument for exploratory health studies. Am J Public Health 1972;62:337–42. 10.2105/AJPH.62.3.337 5011164 PMC1530096

[45] Carney O, McIntosh J, Worth A The use of the Nominal Group Technique in research with community nurses. J Adv Nurs 1996;23:1024–9. 10.1046/j.1365-2648.1996.09623.x 8732532

[46] Bartunek JM, Murningham JK The nominal group technique: expanding the basic procedure and underlying assumptions. Group Organisation Stud 1984;9:417–32. 10.1177/105960118400900307

[47] Ericsson KA, Simon HA Protocol analysis. Verbal reports as data. 1st rev edn Cambridge, MA: MIT Press, 1993.

[48] van Someren MW, Barnard YF, Sandberg JAC The think aloud method. A practical guide to modeling cognitive processses. London: Acadmic Press, 1994.

[49] Jones JA The verbal protocol: a research technique for nursing. J Adv Nurs 1989;14:1062–70. 10.1111/j.1365-2648.1989.tb01518.x 2613960

[50] Aronson J A pragmatic view of thematic analysis: The qualitative report. Secondary A pragmatic view of thematic analysis: The qualitative report 1994. http://www.nova.edu/ssss/QR/BackIssues/QR2–1/aronson.html

[51] Guba E, Lincoln Y Effective evaluation: improving the usefulness of evaluation results through responsive and naturalistic approaches. San Francisco: Jossey-Bass, 1981.

[52] Mays N, Pope C Rigour and qualitative research. BMJ 1995;311:109–12. 10.1136/bmj.311.6997.109 7613363 PMC2550154

[53] Clark D, Centeno C Palliative care in Europe: an emerging approach to comparative analysis. Clin Med 2006;6:197–201. 10.7861/clinmedicine.6-2-197 PMC4953208 16688982

[54] Ahmedzai S, Costa A, Blengini C A new international framework for palliative care. Eur J Cancer 2004;40:2192–200. 10.1016/j.ejca.2004.06.009 15454244

[55] van der Steen JT Dying with dementia: what we know after more than a decade of research. J Alzheimers Dis 2010;22: 37–55. 10.3233/JAD-2010-100744 20847433

